# Ocular Vascular Events following COVID-19 Vaccines: A Systematic Review

**DOI:** 10.3390/vaccines10122143

**Published:** 2022-12-14

**Authors:** Hashem Abu Serhan, Abdelaziz Abdelaal, Mohammad T. Abuawwad, Mohammad J. J. Taha, Sara Irshaidat, Leen Abu Serhan, Luai Abu-Ismail, Qusai Faisal Abu Salim, Basel Abdelazeem, Ayman G. Elnahry

**Affiliations:** 1 Department of Ophthalmology, Hamad Medical Corporations, Doha 3050, Qatar; 2 Tanta Research Team, El-Gharbia 31511, Egypt; 3 Harvard Medical School, Postgraduate Medical Education, Boston, MA 02115, USA; 4 Doheny Eye Institute, University of California, Los Angeles, CA 94720, USA; 5 Department of Clinical Medicine, Kasr Alainy Faculty of Medicine, Cairo University, Cairo 11562, Egypt; 6 Department of Pediatrics, King Hussein Cancer Centre, Amman 11941, Jordan; 7 Faculty of Medicine, Hashemite University, Zarqa 13133, Jordan; 8 Department of Ophthalmology, Islamic Hospital, Amman 11190, Jordan; 9 Department of Ophthalmology, The Eye Specialty Hospital, Amman 11118, Jordan; 10 Department of Internal Medicine, Michigan State University, East Lansing, MI 48824, USA; 11 Department of Ophthalmology, Faculty of Medicine, Cairo University, Cairo 11591, Egypt; 12 Division of Epidemiology and Clinical Applications, National Eye Institute, National Institutes of Health, Bethesda, MD 20892, USA

**Keywords:** vaccination, SARS-CoV-2, ophthalmic adverse events, adverse events, COVID-19 vaccination, vascular events, central artery occlusion, ischemic optic neuropathy

## Abstract

The main aim of this study is to investigate the current evidence regarding the association between COVID-19 vaccination and ocular vascular events. The protocol is registered on PROSPERO (CRD42022358133). On 18 August 2022, an electronic search was conducted through five databases. All original articles reporting individuals who were vaccinated with COVID-19 vaccines and developed ophthalmic vascular events were included. The methodological quality of the included studies was assessed using the NIH tool. A total of 49 studies with 130 ocular vascular cases were included. Venous occlusive events were the most common events (54.3%), which mostly occurred following the first dose (46.2%) and within the first five days following vaccination (46.2%). Vascular events occurred more with the Pfizer and AstraZeneca vaccines (81.6%), and mostly presented unilaterally (73.8%). The most frequently reported treatment was intravitreal anti-VEGF (n = 39, 30.4%). The majority of patients (90.1%) demonstrated either improvement (*p* = 0.321) or persistence (*p* = 0.414) in the final BCVA. Ophthalmic vascular events are serious vision-threatening side effects that have been associated with COVID-19 vaccination. Clinicians should be aware of the possible association between COVID-19 vaccines and ocular vascular events to provide early diagnosis and treatment.

## 1. Introduction

Vaccines against the SARS-CoV-2 infection are the primary modality to prevent the disease from spreading. In 2020, an international race to develop vaccines against SARS-CoV-2 started [1], and by May 2022, a total of nine vaccines had been listed for emergency use by the World Health Organization (WHO): AstraZeneca (recombinant vaccine), Johnson & Johnson/Janssen (recombinant), Pfizer-BioNTech (mRNA), Moderna (mRNA), Sinopharm (inactivated), CoronaVac (inactivated), Novavax (recombinant, adjuvanted), Convidecia (recombinant), and Baharat (inactivated) [2]. Despite substantial protection against severe outcomes following vaccination, and the boosting maintained for most of the population, multiple side effects were reported to occur following vaccination [3]. Generally, WHO defined Adverse Events Following Immunization (AEFI) as any undesirable medical circumstances that occur after vaccination but do not necessarily have a direct link to the use of the vaccine [4]. Regarding COVID-19-vaccine-related complications, vascular complications were the most serious to happen. Many vascular complications of the COVID-19 vaccine were reported including many serious vaccine-related thrombo-embolic events, resulting in cerebral venous thrombosis, thrombocytopenia, and coagulation disorders [5].

Although COVID-19 vaccination can be complicated by several ocular events such as abducens nerve palsy, acute macular neuro-retinopathy, and multiple evanescent white dot syndrome, vascular events remain the most serious group of complications that needs higher medical attention, due to their high association with vision loss and blindness [6]. Despite their rarity, ocular vascular events were indeed reported following COVID-19 vaccines. For example, retinal artery occlusions (RAO), venous stasis retinopathy, and non-arteritic anterior ischemic optic neuropathy (NAAION) were reported in the literature [7]. In early May 2021, The Royal College of Ophthalmologists in the United Kingdom reported an increased incidence of central venous sinus thrombosis (CVST) and retinal vein occlusion (RVO) subsequent to COVID-19 vaccination [8].

Nevertheless, vaccination against COVID-19 is now being conducted on a large scale worldwide due to its proven benefit of preventing severe COVID-19 infection, which is also known to cause vascular events including in the eye [9]. Thus, more light should be shed on the ocular complications generally and vascular events specifically associated with COVID-19 vaccination. In this systematic review, we collect and analyze all observational studies to date that reported cases of ocular vascular events following COVID-19 vaccination, to summarize the current evidence regarding their association. To our knowledge, this is the first systematic review that specifically tackles ocular vascular events occurring after COVID-19 vaccination.

## 2. Materials and Methods

### 2.1. Study Design

This research was conducted in accordance with the Preferred Reporting Items for Systematic Reviews and Meta-Analyses (PRISMA) guidelines, and the protocol was pre-registered on PROSPERO [CRD42022358133]. The design of this research followed the PICOS framework as follows: population (healthy individuals with no prior ocular pathologies), intervention (COVID-19 vaccines of different types and/or doses), comparison (none), outcomes (occurrence of ophthalmic vascular events), and study design (observational and/or experimental studies).

### 2.2. Search Strategy

On 18 August 2022, PubMed, Scopus, Web of Science (WoS), EMBASE, Cochrane Central Register of Controlled Trials (CENTRAL), and Google Scholar were searched for studies reporting the occurrence of ophthalmic vascular events after receiving COVID-19 vaccines. It should be noted that, based on recent recommendations [10], only the first 200 records of Google Scholar were searched, after which their relevance significantly dropped. The following keywords were used to identify relevant articles: (COVID-19 OR SARS-CoV-2) AND (vaccine *) AND (“ophthalmic vascular event *”). Additionally, Medical Subject Headings (MeSH) terms were used to identify all potentially relevant articles based on these indexed terms. The detailed search criteria, adjusted per each searched database, is provided in [Supplementary Table S1].

A manual search was also conducted following the screening of articles to identify any potentially missing relevant articles through three approaches: (a) screening the reference list of included articles, (b) screening “similar articles” to the included ones, through the “similar articles” options on PubMed, and (c) manually searching for articles on Google with the use of following keywords: “COVID” + “vaccine” + “ophthalmic”. The key ophthalmic vascular events that we looked for included choroidal ischemia, retinal artery occlusions (RAO), retinal vein occlusion (RVO), ophthalmic artery occlusion (OAO), ophthalmic vein occlusion (OVO), ophthalmic artery spasm, vitreous hemorrhage, or ischemic optic neuropathy. An updated search was conducted right before the analysis to include any recently published studies in the time between our original and updated search.

### 2.3. Study Outcomes

The primary outcome of this review is to summarize the available evidence on the occurrence of any ophthalmic vascular events following COVID-19 vaccination while providing an emphasis on the association between these events and the type, dose, and time interval from vaccination until their occurrence.

### 2.4. Eligibility Criteria

Studies were included if they recruited individuals who were vaccinated with any of the COVID-19 vaccines and developed an ophthalmic vascular event following vaccination. No limitations were set on language, country, or study design. Of note, case reports, case series, case–control, cohort, cross-sectional, and experimental studies were eligible for inclusion.

On the other hand, studies were excluded if they had one of the following criteria: (1) non-original research (i.e., reviews, commentaries, guidelines, editorials, correspondence, letters to editors, etc.), (2) unavailable full texts, (3) duplicated records or records with overlapping datasets, (4) studies reporting adverse events other than ophthalmic vascular events, and (5) studies that discuss non-COVID-19 vaccines.

### 2.5. Study Selection

Following the retrieval of the studies from the database search, citations were imported into EndNote for duplicate removal, after which, the citations were exported into an Excel Sheet for screening. First, the titles and abstracts of the retrieved articles were screened against our prespecified eligibility criteria. Then, studies that were potentially relevant underwent full-text screenings. This process was carried out by two sets of two reviewers [S.I. and L.A.S.; L.A.I and Q.A.S] who resolved their differences through discussions. Meanwhile, the senior author was consulted when an agreement could not be reached.

### 2.6. Data Extraction

A pilot extraction was carried out to design the data extraction sheet using Microsoft Excel. The data extraction sheet consisted of four main parts. The first part includes the baseline characteristics of the included studies (name of the first author, year of publication, country, name of the journal, and study design) and included participants (sample size, age, and gender). The second part includes data on the reported ophthalmic vascular event (name, type, number, and laterality [right or left eye or both]) and COVID-19 vaccines (type, dose, time from vaccination to symptom onset, and COVID-19 infection status). The third part summarizes the medical history of the reported cases with ophthalmic vascular events (i.e., systemic diseases, cardiovascular diseases, cerebrovascular diseases, immunological diseases, history of eye trauma, previous eye diseases, and previous ocular surgeries). The fourth part included a thorough assessment of the reported event in terms of presenting symptoms, diagnostic methods, examination findings, initial best-corrected visual acuity (BCVA), investigations (blood and eye investigations), management (either medical or surgical), the follow-up period, and management outcomes and associated complications if present. The data extraction process was carried out by two sets of two reviewers [S.I. and L.A.S.; L.A.I and Q.A.S], and any discrepancies were resolved by discussion or consultation with the senior author.

### 2.7. Quality Assessment

The methodological quality of the included studies was assessed using the National Institute of Health (NIH) tool (https://www.nhlbi.nih.gov/health-topics/study-quality-assessment-tools, accessed on 17 October 2022) for each respective study design included (no quality assessment was done for case reports). This process was carried out by two sets of two reviewers [S.I. and L.A.S.; L.A.I and Q.A.S], and any discrepancies were resolved by discussion or consultation with a senior author.

### 2.8. Data Synthesis

Retrieved data from the included studies were qualitatively synthesized. No quantitative analyses were carried out. Frequencies and proportions were used to summarize the data. Comparisons between categorical variables were analyzed using the Pearson Chi-square test. At a *p*-value of 0.05, statistical significance was deemed to exist. The Social Sciences Statistical Program was used to conduct the statistical analysis (IBM SPSS Corp, Statistical Product and Service Solutions (SPSS) Statistics version 26, Chicago, USA). The qualitative synthesis included summarizing the occurrence of ophthalmic vascular events following COVID-19 vaccination, where data were categorized based on the study design and type and dose of the COVID-19 vaccine. Then, our outcome of interest (the occurrence of ophthalmic vascular events) was analyzed in terms of baseline characteristics (age, gender, vaccine type and dose, presenting symptoms, and time interval from vaccination to symptom onset). Such data were stratified by the type and location of the vascular event. Finally, the outcomes of the management of each vascular event were summarized, including complete resolution, partial resolution, recurrence, and complications.

## 3. Results

### 3.1. Search Results

**Figure 1:**
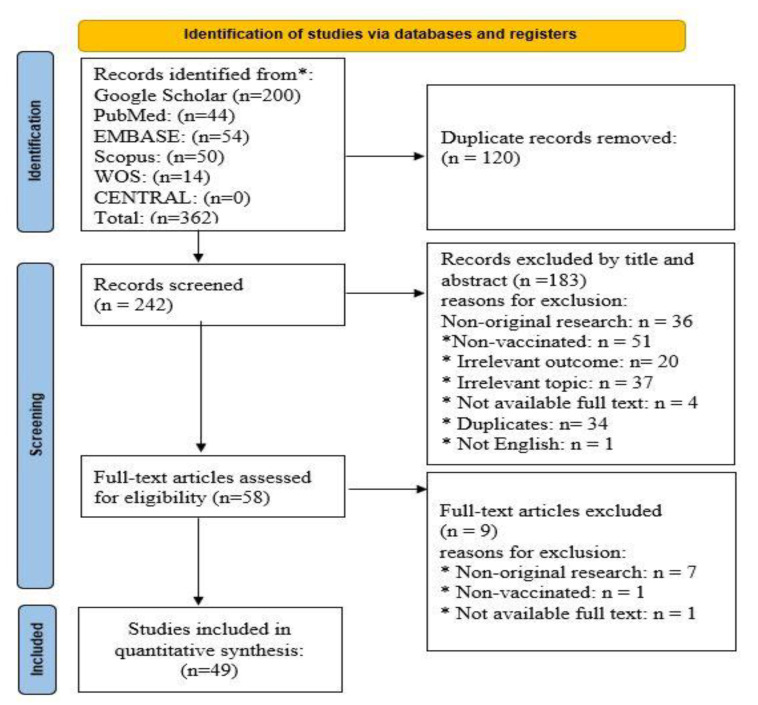
PRISMA chart for article selection. *: the following different databases.

We retrieved 360 records from our searches, 120 duplicates were removed, and the remaining 242 titles and abstracts were screened. Then, 58 potential full texts were assessed and only 49 studies were included (Figure 1). It should be noted that both the manual and updated database search did not yield any additional studies.

### 3.2. Baseline Characteristics of Studies Reporting COVID-19-Vaccine-Associated Vascular Events

In this systematic review, a total of 49 case reports and series with 130 cases of ocular vascular events following COVID-19 vaccination from 23 countries around the world were identified. The included papers are summarized in Table 1.

**Figure 2:**
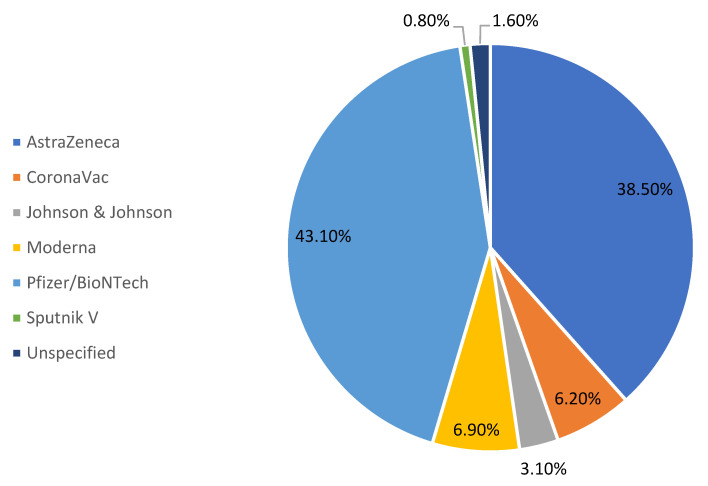
Types of COVID-19 vaccine used in patients with ophthalmic vascular event.

The patients’ ages ranged between 20 and 96, with a mean (±SD) of 58.92 (±17.57), and the population was nearly equally distributed between males and females (51.5%). Pfizer-BioNTech was the most reported vaccine (n = 56, 43.1%), while AstraZeneca was the second most reported with 50 cases (38.5%). The remaining 24 cases (18.6%) were associated with other types of vaccines, namely Moderna, CoronaVac, Johnson & Johnson, one case of non-available data on the vaccine, and one case with a non-specific mRNA vaccine (Figure 2). Regarding the doses, most ocular vascular events occurred after the administration of the first dose (46.2%).

Table 2 shows the demographic characteristics of the included cases, categorized into five main categories: arterial events (CRAO, NAAION, etc.), venous events CRVO/BRVO, etc.), simultaneous arterial and venous together, hemorrhagic events, and other events. Venous events were the most reported events with 69 cases (53%), followed by arterial events with 36 cases (27.7%). There was no significant difference in the five categories regarding age (*p* = 0.692). However, hemorrhagic events were associated mainly with older age (74.15 ± 9.11), while the arterial and venous events were associated with similar age groups (57.86 ± 16.89 and 59.36 ± 16.84 respectively). Regarding gender, all the events were distributed equally in the five categories and we found no statistical difference between them (*p* = 0.804). The AstraZeneca vaccine was associated the most with venous complications (n = 33, 25.4%) compared to the other vaccines, followed by the Pfizer vaccine (n = 27, 20.8%), which was reported the most with arterial complications (*p* = 0.38). The dosage effect was most commonly associated with the first and second doses (88.5%); however, events were evenly distributed between the first and second dosage, except in the dual arterial and venous category, which was mainly associated with the second dose only. The booster dose was reported only in three cases of venous complications (2.3%) (*p* = 0.429).

**Figure 3:**
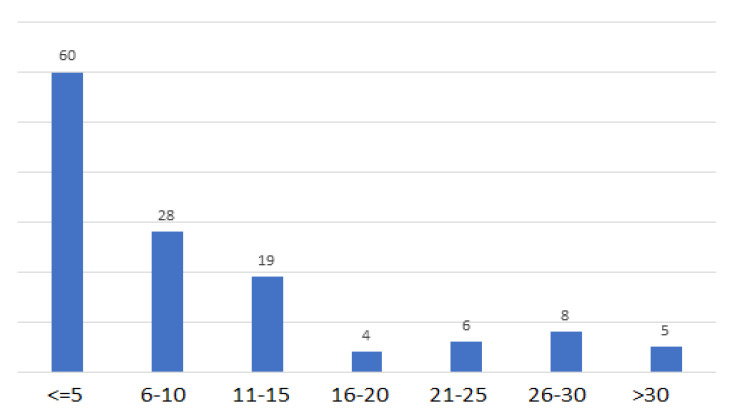
Day of onset of vascular ocular event divided into segments of five days.

Table 3 shows the clinical characteristics of the cases with underlying systemic and ocular diseases. Hypertension was more frequently associated with ocular vascular events compared to diabetes in most of the categories. Furthermore, old vascular events were reported in eight cases, while previous ocular surgeries were reported in 18 cases, and six cases had a history of treatment with anti-vascular endothelial growth factor (VEGF) injections, five of which were associated with hemorrhagic events. In addition, only one case with a history of glaucoma secondary to epiretinal membrane was reported. Regarding the laterality, most cases were unilateral (96 cases, 73.8%) and affected the right eye (*p* = 0.002). As to the duration between vaccination and the ocular events, we classified the durations into five-day categories (Table 3). An inverse relationship was observed between the duration following vaccination and the incidence of ocular vascular events, indicating that most ocular vascular events in this review occurred in the first five days following vaccination (46.2%), which, however, was not statistically significant (*p* = 0.095) (Figure 3). Patients’ complaints were classified into three categories: visual disturbances, non-available data, and others (proptosis, red eye, scalp tenderness, temporal headache, ophthalmoplegia, retrobulbar pain, uveitis, etc.). Visual disturbances included decreased visual acuity, floaters, light flashes, photopsia, curtains obstructing vision, visual field defects, and greyish spots, which represented 68.5% of the total patients’ presenting complaints.

Table 4 shows the interventions that were used in the cases; we classified them into two main groups, medical and surgical. The medical treatment was also subdivided into four groups. Medical treatment was much more common than surgical intervention, as the most frequent treatment used as the first-line therapy following the events was intravitreal anti-VEGF (n = 39, 30.7%), followed by corticosteroids, which were given in 18 (14.2%) of the cases. Nine patients (6.92%) had received some type of thrombolytic, antiplatelet, or anticoagulant, of whom four (3.07%) had received Aspirin, two (1.5%) received Apixaban, one received Clopidogrel, one received Fondaparinux, and one case received a nonspecific anti-platelet. On the other hand, vitrectomy was the most commonly performed surgery (60% of total performed surgeries) (*p* < 0.001). In addition, the use of both intravitreal anti-VEGF and vitrectomy reached a statistically significant point (*p* < 0.001) while other interventions did not. Furthermore, vitrectomy was done almost exclusively for hemorrhagic events (five out of six total), while 76.92% of the total intravitreal anti-VEGF was given after venous vascular complications.

The outcome and degree of improvement of the cases are shown in Table 5 based on the difference between the final BCVA and the initial BCVA, which was calculated using the formula (Final BCVA-Initial BCVA), with any (+) value denoting improvement, any (−) value denoting worsening, and “0” or no change denoting persistence. The improvement was grouped into three categories: improved, persisted, and deteriorated. Among the data that were available, the majority of patients (91.3%) demonstrated either improvement or persistence in the final BCVA. There were no significant differences between improvement, persistence, or worsening between the groups (*p* = 0.369, *p* = 0.516, and *p* = 0.34, respectively). Persistence in venous events was marginally higher than the number of patients who improved, whereas among arterial issues, persistence was more than twice as great as improvement.

Supplementary Table S2 provides an aggregation for all case characteristics and information.

## 4. Discussion

In the present systematic review, 49 reports describing 130 cases of ocular vascular events in close proximity to COVID-19 vaccination were described. This occurred after the first dose or second dose of their Pfizer-BioNTech (n = 56 (43.1%)) or AstraZeneca (n = 50, 38.5%) vaccines. The exact mechanism by which these pathologies occur remains unclear; nevertheless, a few hypotheses were suggested to explain these adverse events. Immune-mediated mechanisms are thought to cause thrombosis through an activation of platelets, immune cells, and hypercoagulability factors [59]. Other potential mechanisms also have been suggested, like molecular mimicry, protein contaminants, and adenovirus vector proteins [60,61]. Since these vascular events are likely brought about by immune-medicated mechanisms, they are more likely to happen after the administration of the first dose due to higher spikes of immunoglobulins after the first exposure, with the risk decreasing with the second and third doses [62]. However, we still identified a relatively large number of cases after the second dose. Although a higher risk of adverse events was attributed to the AstraZeneca vaccine [63], it is hard to validate this information with regards to vascular ocular events since data on vaccine type per population is difficult to acquire. The AstraZeneca vaccine is also reported to be one of the most commonly administered COVID-19 vaccines which may explain its frequent association with adverse events (REF). Most events occurred within five days of vaccination (*p* = 0.095), and 67.8% of events occurred within 10 days post-vaccination. In the literature, retinal vascular events were observed within 3.1 ± 2.4 days of vaccination, and other ocular adverse effects of COVID-19 vaccines generally occurred during the first 10 days after vaccination [64]. This temporal association may be attributed to vaccine-related antibodies that induce hypercoagulability, as they appear within the first 5–10 days after vaccination, and disappear within 100 days [59].

Our cohort had a mean age of 58.92 ± 17.57, falling within the older age group. Age above 50 years was linked to COVID vaccine-related adverse events [39,65], and ocular vascular events were recorded in the same age group in [64]. This was also true when comparing ischemic optic neuropathy versus optic neuritis in patients that developed optic neuropathy after COVID-19 vaccination [61]. Ocular hemorrhagic events were also specifically linked to advanced age [66], which is the case in our population. Older patients (74.2 ± 9.11) had a higher incidence of hemorrhagic vascular complications. This could be attributed to age-related degeneration of macular and choroidal tissues, which may involve neovascularization (NS) and pathologic angiogenesis [67]. Vascular occlusive events of veins (central or branch) were observed with a higher frequency compared to arterial occlusions: 69 venous cases (53%) compared to 36 arterial cases (27.7%). This goes in accordance with observations in the literature, where retinal venous events were observed more than arterial events [68,69,70,71]. The venous involvement in the adverse effects of vaccines is thought to be due to the relation between cerebral veins, including retinal veins, and the clearance of toxins from nasal sinuses, which could lead to higher immunogenicity, hence a higher risk of thrombosis, especially in the setting of immune activation post vaccination [60]. Most patients suffered from a unilateral vascular event, with only nine (6.9%, *p* = 0.002) patients presenting with bilateral ocular affection, as previously observed in literature [70] . The anatomical variations between the right and left retinal veins and arteries can help explain the preferences of retinal vascular events [70]. Given that the majority of cases in this review were of venous occlusion, right eye involvement was higher (n = 58, 44.6%, *p* = 0.002). This can be due to the anatomical relations between the venous system or the right heart and the right eye.

The history of prior underlying systemic diseases was also collected in this study. Hypertension and diabetes were reported more commonly. Overall, hypertension was frequently associated with ocular vascular events in the current review. This is described in the literature as “hypertensive eye disease,” associating chronic and acute elevations in systemic blood pressure with the incidence of ocular vascular events [72]. However, a recent study from Japan suggests that the relationship can be multifactorial and occurs only in females [73]. Changes in systemic blood pressure are directly linked to several ocular complications, since the vasculature of the retina and the optic nerve are vulnerable to fluctuations in blood flow due to limited autoregulation [72]. On the other hand, diabetes compromises retinal blood flow, which in turn predisposes patients to vascular complications [74]. A link between prior intravitreal anti-VEGF injection and hemorrhagic ocular events was also suspected in the current study, since five patients with a history of anti-VEGF treatment presented with hemorrhagic events. This, however, could be a complication of the underlying condition for which the anti-VEGF agent was administered in the first place, or, less likely, a complication related to the anti-VEGF agent’s vascular and inflammatory effects [75,76] . More studies are needed to further evaluate this risk.

In the reviewed cases, a clear management criterion was often not mentioned. Nevertheless, 39 (30.7%, *p* < 0.001) patients received intravitreal anti-VEGF injections of various types, likely because many patients are expected to develop exudative maculopathy following the retinal venous events [77]. The management of vascular ocular events varies between anti-VEGF injections, surgical procedures, steroid therapy, and other medications according to the type of event. In exudative and ischemic events, intravitreal anti-VEGF injections are mostly used [78].

The patients’ improvement was assessed by comparing the patient’s presenting BCVA with the patient’s final BCVA after follow-up and management. Unfortunately, most case reports did not include sufficient data on their management and outcome. The available data showed persisting symptoms in most patients, which is a known feature of most ocular vascular events, although new research suggests long-term improvement [79].

The issue of ocular vascular events as a consequence of COVID-19 vaccination is therefore, arguably, an important cause of blindness for patients that deserves more attention. However, these adverse events are still considered rare based on the millions of vaccine doses administered worldwide. Individuals particularly at risk should be counselled regarding this risk before receiving COVID-19 vaccines particularly because the visual prognosis appears to be guarded. In addition, further research targeting the underlying pathophysiology of these events is required, especially with respect to their risk factors and possible methods of prevention and treatment. Nevertheless, the benefits of COVID-19 vaccination still far outweigh the associated risks. Future case reporting with detailed descriptions of management criteria is needed in order to provide researchers and ophthalmologists with insight on how to treat similar cases.

The limitations of our study include the lack of diagnostic information in many cases, the lack of outcome assessment for the affected eyes in many cases, and the inability to perform relative risk statistical analysis due to insufficient data.

## 5. Conclusions

Ophthalmic vascular events are serious vision-threatening side effects that have been associated with COVID-19 vaccination. We provided the first systematic review dedicated to these events. Luckily, venous occlusive events that are currently most amenable to treatment were the most common among other vascular events. These events occurred after the first and second doses mostly within the first five days following vaccination. Moreover, most events tended to occur in older patients. Further studies are needed to better determine the incidence, risk factors, prognosis, and management of ocular vascular events following COVID-19 vaccination.

## Figures and Tables

**Table 1:** Summary of papers reviewed in this systematic review.

No.	Author	Country	Type of Study	No. of Cases	Mean Age	Gender	Diagnosis
1	Abdallah & Hamzah [11]	USA	Case Report	1	51	M	CRAO
2	Abdin et al. [12]	Germany	Case Report	1	76	F	CRAO
3	Amin et al. [13]	Bangladesh	Case Report	1	41	M	VH
4	Bialasiewicz et al. [14]	Qatar	Case Report	1	50	M	CRVO
5	Bolletta et al. [15]	Italy	Case Series	6	49.5	3 M, 3 F	1 CRVO, 5 BRVO
6	Cackett et al. [16]	UK	Case Report	2	45	2 F	2 CRVO
7	Casarini et al. [17]	Italy	Case Report	1	60	M	VH
8	Che et al. [18]	South Korea	Case Report	1	87	F	AAION
9	Chen et al. [19]	Taiwan	Case Report	1	48	F	BRAO
10	Choi et al. [20]	Korea	Case Series	9	60.8	3 M, 6 F	4 CRVO, 5 BRVO
11	Chow et al. [21]	Taiwan	Case Report	1	70	M	CRAO
12	Chung et al. [22]	Korea	Case Report	1	65	F	NAAION
13	Da Silva et al. [23]	Brazil	Case Series	11	57	3 M, 8 F	5 CRAO, 4 CRVO, 2 Intraretinal Hemorrhage
14	Majumder & Prakash [24]	India	Case Report	1	28	M	CRVO
15	Elhusseiny et al. [25]	USA	Case Report	1	51	M	NAAION
16	Endo et al. [26]	Spain	Case Report	1	52	M	CRVO
17	Franco & Fonollosa [27]	Spain	Case Report	2	59	2 M	2 NAAION
18	Girbardt et al. [7]	India	Case Series	6	46.5	4 M, 2 F	BRAO, CRVO, Venous Stasis Retinopathy, NAAION, CRAO, AMN
19	Goyal et al. [28]	Japan	Case Report	1	28	M	CRVO
20	Ikegami et al. [29]	Japan	Case Report	1	54	F	CRAO
21	Ishibashi et al. [30]	Japan	Case Series	6	59.3	3 M, 3 F	4 BRAO, PAMM, AMN
22	Kang et al. [31]	Korea	Case Report	1	64	M	BRAO
23	Lee et al. [32]	USA	Case Report	1	34	M	CRVO
24	Chen et al. [33]	China	Case Series	5	54.2	4 M, 1 F	BRAO, BRVO, CRAO, CRVO, VH
25	Lin et al. [34]	Taiwan	Case Report	1	61	F	NAAION
26	Maleki et al. [35]	US	Case Series	2	56	2 F	AAION, AZOOR
27	Murgova & Balchev [36]	Bulgaria	Case Series	1	58.4	3 M, 2 F	NAAION
28	Nachbor et al. [37]	Nepal	Case Report	1	64	F	NAAION
29	Nusanti et al. [38]	Indonesia	Case Report	1	50	F	N/A
30	Park et al. [39]	Korea	Case Series	21	77	11 M, 19 F	11 AMD, 10 RVO
31	Peters et al. [40]	Australia	Case Series	5	57	3 M, 2 F	3 BRVO, RVO, CRVO
32	Priluck et al. [41]	USA	Case Report	2	38.5	2 F	BRVO, AMN
33	Pur et al. [42]	Canada	Case Report	1	34	M	BRVO
34	Romano et al. [43]	Italy	Case Report	1	54	F	CRVO
35	Sacconi et al. [44]	Italy	Case Report	1	74	F	RVO
36	Sanjay et al. [45]	India	Case Report	1	50	F	N/A
37	Shah et al. [46]	USA	Case Report	1	27	F	CRVO
38	Sodhi et al. [47]	India	Case Report	1	43	M	CRVO
39	Sonawane et al. [48]	India	Case Report	2	46.5	M	2 CRVO
40	Sugihara et al. [49]	Japan	Case Report	1	38	M	BRVO
41	Takacs et al. [50]	Hungary	Case Report	1	35	M	CRVO
42	Tanaka et al. [51]	Japan	Case Report	2	71.5	M	2 BRVO
43	Suphachaiprasert & Thammakumpee [52]	Thailand	Case Report	1	41	M	CRAO
44	Tsukii et al. [53]	Japan	Case Report	1	55	F	NAAION
45	Vinzamuri et al. [54]	India	Case Report	1	35	M	N/A
46	Vujosevic et al. [55]	Italy	Case Series	14	77	5 M, 9 F	6 BRVO, 6 CRVO, 2 RVO
47	Wang et al. [56]	Taiwan	Case Series	1	47.7	4 M, 7 F	CRAO
48	Elnahry et al. [57]	USA	Case Series	2	50.5	F	NAAION
49	Haseeb et al. [58]	Egypt	Case Report	1	40	M	NAAION

Abbreviations: AAION: Arteritic Anterior Ischemic Optic Neuropathy, AMN: Acute Macular Neuroretinopathy, AZOOR: Acute Zonal Occult Outer Retinopathy, BRVO: Branch Retinal Venous Occlusion, CRAO: Central Retinal Arterial Occlusion, CRVO: Central Retinal Venous Occlusion, NAAION: Non-Arteritic Anterior Ischemic Optic Neuropathy, PAMM: Paracentral Acute Middle Maculopathy, RVO: Retinal Venous Occlusion, VH: Vitreous Hemorrhage.

**Table 2:** Demographic characteristics of the included cases.

Characteristic		Nature of Ocular Event						Total	*p*-Value
		Arterial n (%)		Venous n (%)	Venous & Arterial n (%)	Hemorrhagic n (%)	Others n (%)		
Demographics									
	Age	57.86 ± 16.89		59.39 ± 16.84	56.33 ± 23.58	74.15 ± 9.11	38.33 ± 13.89	58.92 ± 17.57	0.692
	Sex								0.804
		Female	17 (13.1%)	35 (26.9%)	2 (1.5%)	7 (5.4%)	6 (4.6%)	67 (51.5%)	
		Male	19 (14.6%)	34 (26.2%)	1 (0.8%)	6 (4.6%)	3 (2.3%)	63 (48.5%)	
COVID-19 Vaccine									0.380
		AstraZeneca	10 (7.7%)	33 (25.4%)	0 (0%)	3 (2.3%)	4 (3.1%)	50 (38.5%)	
		CoronaVac	4 (3.1%)	2 (1.5%)	0 (0%)	1 (0.8%)	1 (0.8%)	8 (6.2%)	
		Johnson & Johnson	1 (0.8%)	2 (1.5%)	0 (0%)	0 (0%)	1 (0.8%)	4 (3.1%)	
		Moderna	4 (3.1%)	3 (2.3%)	1 (0.8%)	0 (0%)	1 (0.8%)	9 (6.9%)	
		Pfizer-BioNTech	17 (13.1%)	27 (20.8%)	2 (1.5%)	8 (6.2%)	2 (1.5%)	56 (43.1%)	
		Sputnik V	0 (0%)	1 (0.8%)	0 (0%)	0 (0%)	0 (0%)	1 (0.8%)	
		Unspecified	0 (0%)	1 (0.8%)	0 (0%)	1 (0.8%)	0 (0%)	2 (1.6%)	
Dose									0.429
		First	17 (13.1%)	29 (22.3%)	0 (0%)	10 (7.7%)	4 (3.1%)	60 (46.2%)	
		Second	15 (11.5%)	30 (23.1%)	3 (2.3%)	3 (2.3%)	4 (3.1%)	55 (42.3%)	
		Booster	0 (0%)	3 (2.3%)	0 (0%)	0 (0%)	0 (0%)	3 (2.3%)	
		Unspecified	4 (3.1%)	7 (5.4%)	0 (0%)	0 (0%)	1 (0.8%)	12 (9.2%)	
Total			36 (27.7%)	69 (53%)	3 (2.3%)	13 (1%)	9 (4.6%)	130 (100%)	

**Table 3:** Clinical characteristics of included cases.

Characteristic	Nature of Ocular Event						Total	*p*-Value
	Arterial n (%)	Venous n (%)		Venous & Arterial n (%)	Hemorrhagic n (%)	Others n (%)		
	No. of Patients	36 (27.7%)	69 (53%)	3 (2.3%)	13 (1%)	9 (4.6%)	130 (100%)	
Clinical Characteristics								
Underlying Systemic Disease								
	Hypertension	11 (8.7%)	21 (16.5%)	1 (0.8%)	5 (3.9%)	2 (1.6%)	40 (31.5%)	0.964
	Diabetes Mellitus	8 (6.2%)	12 (9.2%)	0 (0%)	6 (4.6%)	0 (0%)	26 (20%)	0.062
	Other	9 (7.2%)	17 (13.6%)	1 (0.8%)	8 (6.4%)	2 (1.6%)	37 (29.6%)	N/A
Underlying Ocular Condition								
	Old Vascular Event	1 (0.8%)	6 (4.8%)	0 (0%)	1 (0.8%)	0 (0%)	8 (6.4%)	0.953
	Old Ocular Surgery/Procedure	4 (3.2%)	9 (7.2%)	0 (0%)	5 (4%)	0 (0%)	18 (14.4%)	0.862
	Anti-VEGF Injections	0 (0%)	1 (0.8%)	0 (0%)	5 (4%)	0 (0%)	6 (4.8%)	0.004
	Other	2 (1.6%)	5 (4%)	0 (0%)	6 (4.7%)	0 (0%)	13 (10.3%)	N/A
Laterality								0.002
	Right	15 (11.5%)	32 (24.6%)	2 (1.5%)	8 (6.2%)	1 (0.8%)	58 (44.6%)	
	Left	11 (8.5%)	19 (14.6%)	1 (0.8%)	4 (3.1%)	3 (2.3%)	38 (29.2%)	
	Bilateral	3 (2.3%)	1 (0.8%)	0 (0%)	1 (0.8%)	4 (3.1%)	9 (6.9%)	
	Not reported	7 (5.4%)	17 (13.1%)	0 (0%)	0 (0%)	1 (0.8%)	25 (19.2%)	
Duration between Vaccination and Ocular Event (days)								0.095
	≤5	17 (13.1%)	33 (25.4%)	1 (0.8%)	6 (4.6%)	3 (2.3%)	60 (46.2%)	
	6–10	6 (4.6%)	16 (12.3%)	0 (0%)	2 (1.5%)	4 (3.1%)	28 (21.5%)	
	11–15	5 (3.8%)	11 (8.5%)	2 (1.5%)	1 (0.8%)	0 (0%)	19 (14.6%)	
	16–20	2 (1.5%)	1 (0.8%)	0 (0%)	1 (0.8%)	0 (0%)	4 (3.1%)	
	21–25	1 (0.8%)	5 (3.8%)	0 (0%)	0 (0%)	0 (0%)	6 (4.6%)	
	26–30	1 (0.8%)	3 (2.3%)	0 (0%)	3 (2.3%)	1 (0.8%)	8 (6.2%)	
	>30	4 (3.1%)	0 (0%)	0 (0%)	0 (0%)	1 (0.8%)	5 (3.8%)	
Main Presenting Complaint								
	Visual Disturbances	26 (20%)	50 (38.5%)	3 (2.3%)	2 (1.5%)	8 (6.2%)	89 (68.5%)	
	Other	2 (1.6%)	2 (1.6%)	0 (0%)	0 (0%)	3 (2.4%)	7 (5.3%)	
	Not reported	10 (7.9%)	19 (15%)	0 (0%)	11 (8.7%)	1 (0.8%)	41 (31.5%)	

**Table 4:** Medical & surgical interventions for included cases.

Management	Nature of Ocular Event						Total	*p*-Value
		Arterial n (%)	Venous n (%)	Venous & Arterial n (%)	Hemorrhagic n (%)	Others n (%)		
Medical								
	Intravitreal anti-VEGF	1 (0.8%)	30 (23.6%)	2 (1.6%)	6 (4.7%)	0 (0%)	39 (30.7%)	<0.001
	Corticosteroid	6 (4.6%)	12 (9.2%)	1 (0.8%)	0 (0%)	1 (0.8%)	20 (15.4%)	0.43
	Observation	3 (2.4%)	9 (7.1%)	1 (0.8%)	2 (1.6%)	1 (0.8%)	16 (12.6%)	0.798
	Other Intervention	7 (5.4%)	4 (3.1%)	0 (0%)	0 (0%)	2 (1.5%)	13 (10%)	0.116
	Unavailable Data	18 (14.2%)	16 (12.6%)	0 (0%)	2 (1.6%)	5 (3.9%)	41 (32.3%)	N/A
Surgical/Procedural								
	Vitrectomy	0 (0%)	1 (0.8%)	0 (0%)	5 (3.9%)	0 (0%)	6 (4.7%)	<0.001
	Laser Procedure	0 (0%)	3 (2.4%)	0 (0%)	0 (0%)	0 (0%)	3 (2.4%)	0.63
	Other Interventions	1 (0.8%)	0 (0%)	0 (0%)	0 (0%)	0 (0%)	1 (0.8%)	0.58
Total		36 (27.7%)	69 (53%)	3 (2.3%)	13 (1%)	9 (4.6%)	130 (100%)	

**Table 5:** The overall outcome for included cases.

Outcome	Nature of Ocular Event					Total	*p*-Value
	Arterial n (%)	Venous n (%)	Venous & Arterial n (%)	Hemorrhagic n (%)	Others n (%)		
Improved	6 (16.7%)	17 (24.6%)	1 (33.3%)	3 (23.1%)	1 (11.1%)	28 (21.5%)	0.369
Persisted	8 (22.2%)	22 (31.9%)	0 (0%)	4 (30.8%)	1 (11.1%)	35 (26.9%)	0.516
Worsened	2 (5.6%)	2 (2.9%)	0 (0%)	2 (15.4%)	0 (0%)	6 (4.6%)	0.34
Unavailable Data	20 (55.6%)	28 (40.6%)	2 (66.7%)	4 (30.8%)	7 (77.8%)	61 (46.9%)	N/A
Total	36 (20%)	69 (41.77%)	3 (1.85%)	13 (7.85%)	9 (5.46%)	130 (100%)	

## Data Availability

The data provided in this manuscript can be provided upon reasonable request by contacting the corresponding author.

## Supplementary Materials

The following supporting information can be downloaded at https://www.mdpi.com/article/10.3390/vaccines10122143/s1, Table S1: The detailed search strategy used in each of the search databases. Table S2: The characteristics and detailed information of all included cases.

## References

[1] Karpiński T.M., Ożarowski M., Seremak-Mrozikiewicz A., Wolski H., Wlodkowic D. (2021). The 2020 race towards SARS-CoV-2 specific vaccines. Theranostics.

[2] WHO (2021). COVID-19 Vaccines with WHO Emergency Use Listing. WHO—Prequalification of Medical Products (IVDs, Medicines, Vaccines and Immunization Devices, Vector Control).

[3] Feikin D.R., Higdon M.M., Abu-Raddad L.J., Andrews N., Araos R., Goldberg Y., Groome M., Huppert A., O'Brien K., Smith P.G. (2022). Duration of effectiveness of vaccines against SARS-CoV-2 infection and COVID-19 disease: Results of a systematic review and meta-regression. Lancet.

[4] WHO (2021). Adverse Events Following Immunization (AEFI).

[5] Pottegård A., Lund L.C., Karlstad Ø., Dahl J., Andersen M., Hallas J., Lidegaard Ø., Tapia G., Gulseth H.L., Ruiz P.L.-D. (2021). Arterial events, venous thromboembolism, thrombocytopenia, and bleeding after vaccination with Oxford-AstraZeneca ChAdOx1-S in Denmark and Norway: Population based cohort study. BMJ.

[6] Taha M.J.J., Abuawwad M.T., Alrubasy W.A., Sameer S.K., Alsafi T., Al-Bustanji Y., Abu-Ismail L., Nashwan A.J. (2022). Ocular manifestations of recent viral pandemics: A literature review. Front. Med..

[7] Girbardt C., Busch C., Al-Sheikh M., Gunzinger J.M., Invernizzi A., Xhepa A., Unterlauft J.D., Rehak M. (2021). Retinal Vascular Events after mRNA and Adenoviral-Vectored COVID-19 Vaccines—A Case Series. Vaccines.

[8] Dean A.G., Arner T.G., Sunki G.G., Friedman R., Lantinga M., Sangam S., Zubieta J.C., Sullivan K.M., Brendel K.A., Gao Z. (2011). Epi Info™, a Database and Statistics Program for Public Health Professionals.

[9] Ritchie H., Mathieu E., Rodés-Guirao L., Appel C., Giattino C., Ortiz-Ospina E., Hasell J., Macdonald B., Beltekian D., Roser M. (2020). Coronavirus pandemic (COVID-19). In *Our World in Data*. https://ourworldindata.org/coronavirus.

[10] Muka T., Glisic M., Milic J., Verhoog S., Bohlius J., Bramer W., Chowdhury R., Franco O.H. (2020). A 24-step guide on how to design, conduct, and successfully publish a systematic review and meta-analysis in medical research. Eur. J. Epidemiol..

[11] Abdallah S., Hamzah K. (2022). Case Report—Central Retinal Artery Occlusion After Ad26.COV2.S COVID-19 Vaccine. Biomed. J. Sci. Tech. Res..

[12] Abdin A.D., Gärtner B.C., Seitz B. (2022). Central retinal artery occlusion following COVID-19 vaccine administration. Am. J. Ophthalmol. Case Rep..

[13] Amin M.A., Nahin S., Dola T.A., Afrin S., Hawlader M.D.H. (2022). Retinal hemorrhage of late post-COVID-19 and post-vaccine-related pathogenic mechanisms: A new challenge for ophthalmologist in COVID era. Clin. Case Rep..

[14] Bialasiewicz A.A., Farah-Diab M.S., Mebarki H.T. (2021). Central retinal vein occlusion occurring immediately after 2nd dose of mRNA SARS-CoV-2 vaccine. Int. Ophthalmol..

[15] Bolletta E., Iannetta D., Mastrofilippo V., De Simone L., Gozzi F., Croci S., Bonacini M., Belloni L., Zerbini A., Adani C. (2021). Uveitis and other ocular complications following COVID-19 vaccination. J. Clin. Med..

[16] Cackett P., Ali A., Young S.L., Pavilion N.L.P.A.E. (2022). Phenotypic appearance of central retinal vein occlusion post AstraZeneca vaccine. Int. J. Ophthalmol..

[17] Casarini B., Bruni F., Rubino P., Mora P. (2022). Vitreous Hemorrhage and Long-Lasting Priapism After COVID-19 m-RNA Based Vaccine: A Case Report. Eur. J. Ophthalmol..

[18] Che S.A., Lee K.Y., Yoo Y.J. (2022). Bilateral Ischemic Optic Neuropathy from Giant Cell Arteritis Following COVID-19 Vaccination. J. Neuro-Ophthalmol..

[19] Chen P.-J., Chang Y.-S., Lim C.-C., Lee Y.-K. (2022). Susac Syndrome Following COVID-19 Vaccination: A Case Report. Vaccines.

[20] Choi M., Seo M.-H., Choi K.-E., Lee S., Choi B., Yun C., Kim S.-W., Kim Y.Y. (2022). Vision-Threatening Ocular Adverse Events after Vaccination against Coronavirus Disease 2019. J. Clin. Med..

[21] Chow S.Y., Hsu Y.-R., Fong V.H. (2022). Central retinal artery occlusion after Moderna mRNA-1273 vaccination. J. Formos. Med. Assoc..

[22] Chung S.A., Yeo S., Sohn S.-Y. (2022). Nonarteritic Anterior Ischemic Optic Neuropathy Following COVID-19 Vaccination: A Case Report. Korean J. Ophthalmol..

[23] Da Silva L.S., Finamor L.P., Andrade G.C., Lima L.H., Zett C., Muccioli C., Sarraf E.P., Marinho P.M., Peruchi J., Oliveira R.D.D.L. (2022). Vascular retinal findings after COVID-19 vaccination in 11 cases: A coincidence or consequence?. Arq. Bras. Oftalmol..

[24] Majumder P.D., Prakash V.J. (2022). Retinal venous occlusion following COVID-19 vaccination: Report of a case after third dose and review of the literature. Indian J. Ophthalmol..

[25] Elhusseiny A.M., Sanders R.N., Siddiqui M.Z., Sallam A.B. (2022). Non-arteritic Anterior Ischemic Optic Neuropathy with Macular Star following COVID-19 Vaccination. Ocul. Immunol. Inflamm..

[26] Endo B., Bahamon S., Martínez-Pulgarín D.F. (2021). Central retinal vein occlusion after mRNA SARS-CoV-2 vaccination: A case report. Indian J. Ophthalmol..

[27] Franco S.V., Fonollosa A. (2022). Ischemic Optic Neuropathy After Administration of a SARS-CoV-2 Vaccine: A Report of 2 Cases. Am. J. Case Rep..

[28] Goyal M., Murthy S., Srinivas Y. (2021). Unilateral retinal vein occlusion in a young, healthy male following Sputnik V vaccination. Indian J. Ophthalmol..

[29] Ikegami Y., Numaga J., Okano N., Fukuda S., Yamamoto H., Terada Y. (2022). Combined central retinal artery and vein occlusion shortly after mRNA-SARS-CoV-2 vaccination. QJM Int. J. Med..

[30] Ishibashi K., Yatsuka H., Haruta M., Kimoto K., Yoshida S., Kubota T. (2022). Branch Retinal Artery Occlusions, Paracentral Acute Middle Maculopathy and Acute Macular Neuroretinopathy After COVID-19 Vaccinations. Clin. Ophthalmol..

[31] Kang M.S., Kim S.Y., Kwon H.J. (2022). Case Report: Recanalization of Branch Retinal Artery Occlusion Due to Microthrombi Following the First Dose of SARS-CoV-2 mRNA Vaccination. Front. Pharmacol..

[32] Lee S., Sankhala K.K., Bose S., Gallemore R.P. (2022). Combined Central Retinal Artery and Vein Occlusion with Ischemic Optic Neuropathy After COVID-19 Vaccination. Int. Med. Case Rep. J..

[33] Chen X., Li X., Li H., Li M., Gong S. (2022). Ocular Adverse Events after Inactivated COVID-19 Vaccination in Xiamen. Vaccines.

[34] Lin W.-Y., Wang J.-J., Lai C.-H. (2022). Non-Arteritic Anterior Ischemic Optic Neuropathy Following COVID-19 Vaccination. Vaccines.

[35] Maleki A., Look-Why S., Manhapra A., Foster C.S. (2021). COVID-19 recombinant mRNA vaccines and serious ocular inflammatory side effects: Real or coincidence?. J. Ophthalmic Vis. Res..

[36] Murgova S., Balchev G. (2022). Ophthalmic manifestation after SARS-CoV-2 vaccination: A case series. J. Ophthalmic Inflamm. Infect..

[37] Nachbor K.M., Naravane A.V., Adams O.E., Abel A.S. (2021). Nonarteritic anterior ischemic optic neuropathy associated with COVID-19 vaccination. J. Neuroophthalmol..

[38] Nusanti S., Putera I., Sidik M., Edwar L., Koesnoe S., Rachman A., Kurniawan M., Tarigan T.J.E., Yunus R.E., Saraswati I. (2022). A case of aseptic bilateral cavernous sinus thrombosis following a recent inactivated SARS-CoV-2 vaccination. Taiwan J. Ophthalmol..

[39] Park H.S., Byun Y., Byeon S.H., Kim S.S., Kim Y.J., Lee C.S. (2021). Retinal hemorrhage after SARS-CoV-2 vaccination. J. Clin. Med..

[40] Peters M.C., Cheng S.S.H., Sharma A., Moloney T.P., Franzco S.S.H.C., Franzco A.S., Franzco T.P.M. (2022). Retinal vein occlusion following COVID-19 vaccination. Clin. Exp. Ophthalmol..

[41] Priluck A.Z., Arevalo J.F., Pandit R.R. (2022). Ischemic retinal events after COVID-19 vaccination. Am. J. Ophthalmol. Case Rep..

[42] Pur D.R., Bursztyn L.L.C.D., Iordanous Y. (2022). Branch retinal vein occlusion in a healthy young man following mRNA COVID-19 vaccination. Am. J. Ophthalmol. Case Rep..

[43] Romano D., Morescalchi F., Romano V., Semeraro F. (2022). COVID-19 AdenoviralVector Vaccine and Central Retinal Vein Occlusion. Ocul. Immunol. Inflamm..

[44] Sacconi R., Simona F., Forte P., Querques G. (2022). Retinal vein occlusion following two doses of mRNA-1237 (moderna) immunization for SARS-CoV-2: A case report. Ophthalmol. Ther..

[45] Sanjay S., Acharya I., Rawoof A., Shetty R. (2022). Non-arteritic anterior ischaemic optic neuropathy (NA-AION) and COVID-19 vaccination. BMJ Case Rep..

[46] Shah P.P., Gelnick S., Jonisch J., Verma R. (2021). Central Retinal Vein Occlusion Following BNT162b2 (Pfizer-BioNTech) COVID-19 Messenger RNA Vaccine. Retin. Cases Brief Rep..

[47] Sodhi P.K., Yadav A., Sharma B., Sharma A., Kumar P. (2022). Central Retinal Vein Occlusion Following the First Dose of COVID Vaccine. Cureus.

[48] Sonawane N., Yadav D., Kota A., Singh H. (2022). Central retinal vein occlusion post-COVID-19 vaccination. Indian J. Ophthalmol..

[49] Sugihara K., Kono M., Tanito M. (2022). Branch Retinal Vein Occlusion after Messenger RNA-Based COVID-19 Vaccine. Case Rep. Ophthalmol..

[50] Takacs A., Ecsedy M., Nagy Z.Z. (2022). Possible COVID-19 MRNA Vaccine-Induced Case of Unilateral Central Retinal Vein Occlusion. Ocul. Immunol. Inflamm..

[51] Tanaka H., Nagasato D., Nakakura S., Tanabe H., Nagasawa T., Wakuda H., Imada Y., Mitamura Y., Tabuchi H. (2021). Exacerbation of branch retinal vein occlusion post SARS-CoV2 vaccination. Medicine.

[52] Suphachaiprasert K.T., Thammakumpee K. (2022). A Cilioretinal Artery Occlusion (CLRAO) Associated with Optic Disc Edema after Viral Vector SARS-CoV-2 Vaccination: Case Report. J. Med. Assoc. Thail..

[53] Tsukii R., Kasuya Y., Makino S. (2021). Nonarteritic anterior ischemic optic neuropathy following COVID-19 vaccination: Consequence or coincidence. Case Rep. Ophthalmol. Med..

[54] Kotian R., Vinzamuri S., Pradeep T. (2021). Bilateral paracentral acute middle maculopathy and acute macular neuroretinopathy following COVID-19 vaccination. Indian J. Ophthalmol..

[55] Vujosevic S., Limoli C., Romano S., Vitale L., Villani E., Nucci P. (2022). Retinal vascular occlusion and SARS-CoV-2 vaccination. Graefe’s Arch. Clin. Exp. Ophthalmol..

[56] Hsu Y.-R., Wang L.-U., Chen F.-T., Wang J.-K., Huang T.-L., Chang P.-Y., Chen Y.-J. (2022). Ocular inflammatory manifestations following COVID-19 vaccinations in Taiwan: A case series. Taiwan J. Ophthalmol..

[57] Elnahry A.G., Asal Z.B., Shaikh N., Dennett K., Abd Elmohsen M.N., Elnahry G.A., Shehab A., Vytopil M., Ghaffari L., Athappilly G.K. (2021). Optic neuropathy after COVID-19 vaccination: A report of two cases. Int. J. Neurosci..

[58] Haseeb A., Elhusseiny A.M., Chauhan M.Z., Elnahry A.G. (2022). Optic neuropathy after COVID-19 vaccination: Case report and systematic review. Neuroimmunol. Rep..

[59] Bilotta C., Perrone G., Adelfio V., Spatola G.F., Uzzo M.L., Argo A., Zerbo S. (2021). COVID-19 Vaccine-Related Thrombosis: A Systematic Review and Exploratory Analysis. Front. Immunol..

[60] McGonagle D., De Marco G., Bridgewood C. (2021). Mechanisms of immunothrombosis in vaccine-induced thrombotic thrombocytopenia (VITT) compared to natural SARS-CoV-2 infection. J. Autoimmun..

[61] Elnahry A.G., Al-Nawaflh M.Y., Eldin A.A.G., Solyman O., Sallam A.B., Phillips P.H., Elhusseiny A.M. (2022). COVID-19 Vaccine-Associated Optic Neuropathy: A Systematic Review of 45 Patients. Vaccines.

[62] Simpson C.R., Shi T., Vasileiou E., Katikireddi S.V., Kerr S., Moore E., McCowan C., Agrawal U., Shah S.A., Ritchie L.D. (2021). First-dose ChAdOx1 and BNT162b2 COVID-19 vaccines and thrombocytopenic, thromboembolic and hemorrhagic events in Scotland. Nat. Med..

[63] Ostrowski S.R., Søgaard O.S., Tolstrup M., Stærke N.B., Lundgren J., Østergaard L., Hvas A.M. (2021). Inflammation and platelet activation after COVID-19 vaccines-possible mechanisms behind vaccine-induced immune thrombocytopenia and thrombosis. Front. Immunol..

[64] Haseeb A.A., Solyman O., Abushanab M.M., Obaia A.S.A., Elhusseiny A.M. (2022). Ocular Complications Following Vaccination for COVID-19: A One-Year Retrospective. Vaccines.

[65] Vo A.D., La J., Wu J.T.Y., Strymish J.M., Ronan M., Brophy M., Do N.V., Branch-Elliman W., Fillmore N.R., Monach P.A. (2022). Factors Associated with Severe COVID-19 Among Vaccinated Adults Treated in US Veterans Affairs Hospitals. JAMA Netw. Open.

[66] Jiang H., Gao Y., Fu W., Xu H. (2022). Risk Factors and Treatments of Suprachoroidal Hemorrhage. BioMed Res. Int..

[67] Thomas C.J., Mirza R.G., Gill M.K. (2021). Age-Related Macular Degeneration. Med. Clin. N. Am..

[68] Chang Y.-S., Ho C.-H., Chu C.-C., Wang J.-J., Tseng S.-H., Jan R.-L. (2018). Risk of retinal artery occlusion in patients with diabetes mellitus: A retrospective large-scale cohort study. PLoS ONE.

[69] Chang Y.S., Jan R.L., Weng S.F., Wang J.J., Chio C.C., Wei F.T., Chu C.C. (2012). Retinal artery occlusion and the 3-year risk of stroke in Taiwan: A nationwide population-based study. Am. J. Ophthalmol..

[70] Li Y., Hall N.E., Pershing S., Hyman L., Haller J.A., Lee A.Y., Lee C.S., Chiang M., Lum F., Miller J.W. (2021). Age, Gender, and Laterality of Retinal Vascular Occlusion: A Retrospective Study from the IRIS^®^ Registry. Ophthalmol. Retin..

[71] Song P., Xu Y., Zha M., Zhang Y., Rudan I. (2019). Global epidemiology of retinal vein occlusion: A systematic review and meta-analysis of prevalence, incidence, and risk factors. J. Glob. Health.

[72] Cheung C.Y., Biousse V., Keane P.A., Schiffrin E.L., Wong T.Y. (2022). Hypertensive eye disease. Nat. Rev. Dis. Prim..

[73] Kinouchi R., Ishiko S., Hanada K., Hayashi H., Mikami D., Yoshida A. (2021). Identification of risk factors for retinal vascular events in a population-based cross-sectional study in Rumoi, Japan. Sci. Rep..

[74] Campochiaro P.A. (2015). Molecular pathogenesis of retinal and choroidal vascular diseases. Prog. Retin. Eye Res..

[75] Porta M., Striglia E. (2019). Intravitreal anti-VEGF agents and cardiovascular risk. Intern. Emerg. Med..

[76] Anderson W.J., da Cruz N.F.S., Lima L.H., Emerson G.G., Rodrigues E.B., Melo G.B. (2021). Mechanisms of sterile inflammation after intravitreal injection of antiangiogenic drugs: A narrative review. Int. J. Retin. Vitr..

[77] Marín-Lambíes C., Gallego-Pinazo R., Salom D., Navarrete-Sanchis J., Díaz-Llopis M. (2012). Rapid Regression of Exudative Maculopathy in Idiopathic Retinitis, Vasculitis, Aneurysms and Neuroretinitis Syndrome after Intravitreal Ranibizumab. Case Rep. Ophthalmol..

[78] Schmidt-Erfurth U., Garcia-Arumi J., Gerendas B.S., Midena E., Sivaprasad S., Tadayoni R., Wolf S., Loewenstein A. (2019). Guidelines for the Management of Retinal Vein Occlusion by the European Society of Retina Specialists (EURETINA). Ophthalmologica.

[79] Scott I.U., VanVeldhuisen P.C., Oden N.L., Ip M.S., Blodi B.A. (2022). Month 60 Outcomes After Treatment Initiation with Anti–Vascular Endothelial Growth Factor Therapy for Macular Edema Due to Central Retinal or Hemiretinal Vein Occlusion. Am. J. Ophthalmol..

