# Supplementary material for: Ocular Vascular Events following COVID-19 Vaccines: A Systematic Review
Source: Vaccines (Basel). 2022 Dec 14;10(12):2143. doi: 10.3390/vaccines10122143 (PMC9786009; doi:10.3390/vaccines10122143)
Supplement: Supplementary file 1 [file vaccines-10-02143-s001.zip › vaccines-2074694-supplementary.pdf]

Supplementary Table S1. The detailed search strategy used in each of the search databases

Database	No.	Search Query	Results
**PubMed [Date of search: August 18, 2022]**			
	#1	("COVID-19"[Mesh] OR "SARS-CoV-2"[Mesh] OR 2019-ncov*[tiab] OR 2019ncov*[tiab] OR 2019-novel-cov*[tiab] OR coronavirus-2*[tiab] OR coronavirus-disease-19*[tiab] OR corona-virus-disease-19*[tiab] OR coronavirus-disease-20*[tiab] OR corona-virus-disease-20*[tiab] OR covid-19*[tiab] OR covid19*[tiab] OR covid-20*[tiab] OR covid20*[tiab] OR ncov-2019*[tiab] OR ncov2019*[tiab] OR new-coronavirus[tiab] OR new-corona-virus[tiab] OR novel-coronavirus[tiab] OR novel-corona-virus[tiab] OR sars-2*[tiab] OR sars2*[tiab] OR sars-cov-19*[tiab] OR sars-cov19*[tiab] OR sarscov19*[tiab] OR sarscov-19*[tiab] OR sars-cov-2*[tiab] OR sars-cov2*[tiab] OR sarscov2*[tiab] OR sarscov-2*[tiab] OR (("Coronavirus"[mh] OR "Coronavirus Infections"[mh] OR betacoronavirus[tiab] OR beta-coronavirus[tiab] OR beta-corona-virus[tiab] OR corona-virus[tiab] OR coronavirus[tiab] OR sars*[tiab] OR severe-acute-respiratory*[tiab]) AND (2019[tiab] OR 2020[tiab] OR wuhan*[tiab] OR hubei*[tiab] OR china*[tiab] OR chinese*[tiab] OR outbreak*[tiab] OR epidemic*[tiab] OR pandemic*[tiab]))) AND 2019/12:3000[dp]	283745
	#2	Pfizer-BioNTech OR BTN162b2 OR Sinopharm OR Sinovac OR Moderna OR AstraZeneca OR ChAdOx1 OR AZD1222 OR Janssen OR "Johnson & Johnson" OR Novavax OR CoronaVac OR Covaxin OR Convidecia OR Sputnik OR Zifivax OR Corbevax OR COVIran OR SCB-2019 OR vaccin* OR "COVID-19 Vaccines"[Mesh]	568389
	#3	"choroidal ischemia" OR "retinal artery occlusion" OR "retinal vein occlusion" OR "ophthalmic artery occlusion" OR " ophthalmic vein occlusion" OR "ophthalmic artery spasm" OR "vitreous hemorrhage" OR "ischemic optic neuropathy" OR "Retinal Artery Occlusion"[Mesh] OR "Retinal Vein Occlusion"[Mesh] OR "Vitreous Hemorrhage"[Mesh] OR "Optic Neuropathy, Ischemic"[Mesh]	16375
	#4	#1 AND #2 AND #3	43
**Scopus [Date of search: August 18, 2022]**			
	#1	(TITLE-ABS-KEY (covid-19) OR TITLE-ABS-KEY (sars-cov-2) OR TITLE-ABS-KEY (2019-ncov*) OR TITLE-ABS-KEY (2019ncov*) OR TITLE-ABS-KEY (2019-novel-cov*) OR TITLE-ABS-KEY (coronavirus-2*) OR TITLE-ABS-KEY (coronavirus-disease-19*) OR TITLE-ABS-KEY (corona-virus-disease-19*) OR TITLE-ABS-KEY (coronavirus-disease-20*) OR TITLE-ABS-KEY (corona-virus-disease-20*) OR TITLE-ABS-KEY (covid-19*) OR TITLE-ABS-KEY (covid19*) OR TITLE-ABS-KEY (covid-20*) OR TITLE-ABS-KEY (covid20*) OR TITLE-ABS-KEY (ncov-2019*) OR TITLE-ABS-KEY (ncov2019*) OR TITLE-ABS-KEY (new-coronavirus) OR TITLE-ABS-KEY (new-corona-virus) OR TITLE-ABS-KEY (novel-coronavirus) OR TITLE-ABS-KEY (novel-corona-virus) OR TITLE-ABS-KEY (sars-2*) OR TITLE-ABS-KEY (sars2*) OR TITLE-ABS-KEY (sars-cov-19*) OR TITLE-ABS-KEY (sars-cov19) OR TITLE-ABS-KEY (sarscov19*) OR TITLE-ABS-KEY (sarscov-19*) OR TITLE-ABS-KEY (sars-cov-2*) OR TITLE-ABS-KEY (sars-cov2*) OR TITLE-ABS-KEY (sarscov2*) OR TITLE-ABS-KEY (sarscov-2*) OR TITLE-ABS-KEY (coronavirus) OR TITLE-ABS-KEY (coronavirus AND infections) OR TITLE-ABS-KEY (betacoronavirus) OR TITLE-ABS-KEY (beta-coronavirus) OR TITLE-ABS-KEY (beta-corona-virus) OR TITLE-ABS-KEY (corona-virus) OR TITLE-ABS-KEY (coronavirus) OR TITLE-ABS-KEY (sars*))	423774
	#2	(TITLE-ABS-KEY (pfizer-biontech) OR TITLE-ABS-KEY (btn162b2) OR TITLE-ABS-KEY (sinopharm) OR TITLE-ABS-KEY (sinovac) OR TITLE-ABS-KEY (moderna) OR TITLE-ABS-KEY (astrazeneca) OR TITLE-ABS-KEY (chadox1) OR TITLE-ABS-KEY (azd1222) OR TITLE-ABS-KEY (janssen) OR TITLE-ABS-KEY (johnson AND & AND johnson) OR TITLE-ABS-KEY (novavax) OR TITLE-ABS-KEY (coronavac) OR TITLE-ABS-KEY (covaxin) OR TITLE-ABS-KEY (convidecia) OR TITLE-ABS-KEY (sputnik) OR TITLE-ABS-KEY (zifivax) OR TITLE-ABS-KEY (corbevax) OR TITLE-ABS-KEY (coviran) OR TITLE-ABS-KEY (scb-2019) OR TITLE-ABS-KEY (vaccin*) OR TITLE-ABS-KEY (covid-19 AND vaccines))	662266

	#3	(TITLE-ABS-KEY (choroidal AND ischemia) OR TITLE-ABS-KEY (retinal AND artery AND occlusion) OR TITLE-ABS-KEY (retinal AND vein AND occlusion) OR TITLE-ABS-KEY (ophthalmic AND artery AND occlusion) OR TITLE-ABS-KEY (ophthalmic AND vein AND occlusion) OR TITLE-ABS-KEY (ophthalmic AND artery AND spasm) OR TITLE-ABS-KEY (vitreous AND hemorrhage) OR TITLE-ABS-KEY (ischemic AND optic AND	32070
	#4	#1 AND #2 AND #3	49
**EMBASE [Date of search: August 18, 2022]**			
	#1	covid 19':ti,ab,kw OR 'sars cov 2':ti,ab,kw OR '2019 ncov':ti,ab,kw OR 2019ncov:ti,ab,kw OR '2019 novel cov':ti,ab,kw OR 'coronavirus2':ti,ab,kw OR 'coronavirus disease 19*':ti,ab,kw OR 'corona virus disease 19*':ti,ab,kw OR 'coronavirus disease 20*':ti,ab,kw OR 'corona virus disease 20*':ti,ab,kw OR 'covid 19*':ti,ab,kw OR covid19*:ti,ab,kw OR 'covid20*':ti,ab,kw OR covid20*:ti,ab,kw OR 'ncov 2019*':ti,ab,kw OR ncov2019*:ti,ab,kw OR 'new coronavirus':ti,ab,kw OR 'new corona virus':ti,ab,kw OR 'novel coronavirus':ti,ab,kw OR 'novel corona virus':ti,ab,kw OR 'sars 2*':ti,ab,kw OR sars2*:ti,ab,kw OR 'sars cov 19*':ti,ab,kw OR 'sars cov19*':ti,ab,kw OR sarscov19*:ti,ab,kw OR 'sarscov 19*':ti,ab,kw OR 'sars cov2*':ti,ab,kw OR sarscov2*:ti,ab,kw OR 'sarscov 2*':ti,ab,kw OR 'coronavirus infections':ti,ab,kw OR betacoronavirus:ti,ab,kw OR 'beta coronavirus':ti,ab,kw OR 'beta corona virus':ti,ab,kw OR 'corona virus':ti,ab,kw OR coronavirus:ti,ab,kw	310184
	#2	coronavirus disease 2019'/exp OR 'coronavirusdisease 2019' OR 'severe acute respiratory syndrome coronavirus 2'/exp OR 'severe acute respiratory syndrome coronavirus 2' OR 'coronavirinae'/exp OR 'coronavirinae'	295063
	#3	pfizer biontech':ti,ab,kw OR btn162b2:ti,ab,kw OR sinopharm:ti,ab,kw OR sinovac:ti,ab,kw OR moderna:ti,ab,kw OR astrazeneca:ti,ab,kw OR chadox1:ti,ab,kw OR azd1222:ti,ab,kw OR janssen:ti,ab,kw OR 'johnson & johnson':ti,ab,kw OR novavax:ti,ab,kw OR coronavac:ti,ab,kw OR covaxin:ti,ab,kw OR convidecia:ti,ab,kw OR sputnik:ti,ab,kw OR zifivax:ti,ab,kw OR corbevax:ti,ab,kw OR coviran:ti,ab,kw OR 'scb 2019':ti,ab,kw OR vaccin*:ti,ab,kw OR 'covid-19 vaccines':ti,ab,kw	495148
	#4	sars-cov-2 vaccine'/exp OR 'sars-cov-2 vaccine' OR 'pfizer biontech'/exp OR 'pfizer biontech' OR 'covilo'/exp OR 'covilo' OR 'coronavac'/exp OR 'coronavac' OR 'elasomeran'/exp OR 'elasomeran' OR 'vaxzevria'/exp OR 'vaxzevria' OR 'ad26.cov2.s vaccine'/exp OR 'ad26.cov2.s vaccine' OR 'nvx-cov2373 vaccine'/exp OR 'nvx-cov2373 vaccine' OR 'covaxin'/exp OR 'covaxin' OR 'convidecia'/exp OR 'convidecia' OR 'zifivax'/exp OR 'zifivax' OR 'corbevax'/exp OR 'corbevax' OR 'coviran barekat'/exp OR 'coviran barekat'	23455
	#5	#1 OR #2	342318
	#6	#3 OR #4	497965
	#7	choroidal ischemia':ti,ab,kw OR 'retinal artery occlusion':ti,ab,kw OR 'ophthalmic artery occlusion':ti,ab,kw OR 'ophthalmic vein occlusion':ti,ab,kw OR 'ophthalmic artery spasm':ti,ab,kw OR 'ischemic optic neuropathy':ti,ab,kw OR 'retina artery occlusion':ti,ab,kw OR 'retina vein occlusion':ti,ab,kw OR 'vitreous hemorrhage':ti,ab,kw OR 'optic neuropathy, ischemic':ti,ab,kw	10330
	#8	choroidal ischemia'/exp OR 'ophthalmic artery occlusion'/exp OR 'ophthalmic vein occlusion' OR 'retina artery occlusion'/exp OR 'retina vein occlusion'/exp OR 'vitreous hemorrhage'/exp OR 'ischemic optic neuropathy'/exp	26233
	#9	#7 OR #8	28441
	#10	#5 AND #6 AND #9	54
**WOS [Date of search: August 18, 2022]**			
	#1	COVID-19 (All Fields) or SARS-CoV-2 (All Fields) or 2019-ncov* (All Fields) or 2019ncov* (All Fields) or 2019-novel-cov* (All Fields) or coronavirus-2* (All Fields) or coronavirus-disease-19* (All Fields) or corona-virus-disease-19* (All Fields) or oronavirus-disease-20* (All Fields) or corona-virus-disease-20* (All Fields) or covid-19* (All Fields) or covid19* (All Fields) or covid-20* (All Fields) or covid20* (All Fields) or ncov-2019* (All Fields) or ncov2019* (All Fields) or new-coronavirus (All Fields) or new-corona-virus (All Fields) or novel-coronavirus (All Fields) or novel-corona-virus (All Fields) or sars-2* (All Fields) or sars2* (All Fields) or sars-	379947

		cov-19* (All Fields) or sars-cov19* (All Fields) or sarscov19* (All Fields) or sarscov-19* (All Fields) or sars-cov-2* (All Fields) or sars-cov2* (All Fields) or sarscov2* (All Fields) or sarscov-2* (All Fields) or Coronavirus (All Fields) or Coronavirus Infections (All Fields) or betacoronavirus (All Fields) or beta-coronavirus (All Fields) or beta-corona-virus (All Fields) or corona-virus (All Fields) or coronavirus (All Fields) or sars* (All Fields)	
	#2	PfizerBioNTech (All Fields) or BTN162b2 (All Fields) or Sinopharm (All Fields) or Sinovac (All Fields) or Moderna (All Fields) or AstraZeneca (All Fields) or ChAdOx1 (All Fields) or AZD1222 (All Fields) or Janssen (All Fields) or Johnson Johnson (All Fields) or Novavax (All Fields) or CoronaVac (All Fields) or Covaxin (All Fields) or Convidecia (All Fields) or Sputnik (All Fields) or Zifivax (All Fields) or Corbevax (All Fields) or COVIran (All Fields) or SCB-2019 (All Fields) or vaccin (All Fields) or COVID19 Vaccines (All Fields)	768044
	#3	choroidal ischemia (All Fields) or retinal artery occlusion (All Fields) or retinal vein occlusion (All Fields) or ophthalmic artery occlusion (All Fields) or ophthalmic vein occlusion (All Fields) or ophthalmic artery spasm (All Fields) or vitreous hemorrhage (All Fields) or ischemic optic neuropathy (All Fields) or Retinal Artery Occlusion (All Fields) or Retinal Vein Occlusion (All Fields) or Vitreous Hemorrhage (All Fields) or Optic Neuropathy, Ischemic (All Fields)	18478
	#4	#1 AND #2 AND #3	14
**CENTRAL [Date of search: August 18, 2022]**			
	#1	(COVID-19):ti,ab,kw OR (SARS-CoV-2):ti,ab,kw OR ("coronavirus infection"):ti,ab,kw OR (novel-coronavirus):ti,ab,kw OR (sars-2):ti,ab,kw	11678
	#2	(Coronavirus Infections):ti,ab,kw OR (betacoronavirus):ti,ab,kw AND (covid20):ti,ab,kw AND (new-coronavirus):ti,ab,kw AND (sarscov2):ti,ab,kw	3251
	#3	#1 OR #2	11770
	#4	(Pfizer-BioNTech):ti,ab,kw OR (BTN162b2):ti,ab,kw OR (Sinopharm):ti,ab,kw OR (Sinovac):ti,ab,kw OR (Moderna):ti,ab,kw (Word variations have been searched)	348
	#5	(AstraZeneca):ti,ab,kw OR (ChAdOx1):ti,ab,kw OR (AZD1222):ti,ab,kw OR (Janssen):ti,ab,kw OR (Johnson & Johnson):ti,ab,kw	3825
	#6	(Novavax):ti,ab,kw OR (CoronaVac):ti,ab,kw OR (Covaxin):ti,ab,kw OR (Convidecia):ti,ab,kw OR (Sputnik):ti,ab,kw	108
	#7	(Zifivax):ti,ab,kw OR (Corbevax):ti,ab,kw OR (COVIran):ti,ab,kw OR (SCB-2019):ti,ab,kw OR (COVID-19 Vaccines):ti,ab,kw (Word variations have been searched)	1500
	#8	#4 OR #5 OR #6 OR #7	5378
	#9	(choroidal ischemia):ti,ab,kw OR (retinal artery occlusion):ti,ab,kw OR (retinal vein occlusion):ti,ab,kw OR (ophthalmic artery occlusion):ti,ab,kw OR (ophthalmic vein occlusion):ti,ab,kw	1125
	#10	(ophthalmic artery spasm):ti,ab,kw OR (vitreous hemorrhage):ti,ab,kw OR (ischemic optic neuropathy):ti,ab,kw OR (Retinal Artery Occlusion):ti,ab,kw OR ("retinal vein occlusion"):ti,ab,kw	1809
	#11	#9 OR #10	1864
	#12	#3 AND #8 AND #11	0
**Google Scholar [Date of search: August 18, 2022]**			
	With all of the words	COVID vaccin	

With the exact phrase	
With at least one of the words	"ischemic optic neuropathy" "choroidal ischemia" "retinal artery occlusion" "retinal vein occlusion" "ophthalmic artery occlusion" "ophthalmic vein occlusion" "ophthalmic artery spasm" "vitreous hemorrhage"
Total	200

[Supplementary Table S2] The characteristics and detailed information of all included cases.

Source	Age	Gender	Country	Vaccination		History (Hx)	Clinical Characteristics					Management		Final BCVA	Outcome	Diagnosis
				Vaccine	Dose	General Hx	Ocular Hx	Onset	Main Complaint	Laterality	Presentation BCVA (Snellen)	Medical	Surgical			
**Abdallah & Hamzah**	51	M	USA	Johnson & Johnson	N/A			4	painless loss of vision	R	HM			N/A	N/A	CRAO
**Abdin et al.**	76	F	Germany	AstraZeneca	1	Hypothyroidism		2	painless loss of vision	L	HM			N/A	N/A	CRAO
**Amin et al.**	41	M	Bangladesh	AstraZeneca	2	HTN		43	floaters in his right eye, e light flashes, a curtain moving into and obstructing his vision, and diminishe	R	N/A			N/A	N/A	VH

									d vision in his right eye and became red in color on right eye							
**Bialasiewicz et al.**	50	M	Qatar	Pfizer/BioNTech	2	Atopic Dermatitis		15 min	retrobulbar pain, red eye and vision reduction on his left eye.	L	N/A			N/A	N/A	CRVO
**Bolletta et al.**	39	M	Italy	Moderna	2			30	decreased VA	NA	1.30	intravitreal anti-VEGF		0.70	I	CRVO
**Bolletta et al.**	53	F	Italy	AstraZeneca	2	HTN		2	decreased VA	NA	0.70	intravitreal anti-VEGF		0.30	I	BRVO
**Bolletta et al.**	61	F	Italy	AstraZeneca	2			2	decreased VA	NA	0.20	intravitreal anti-VEGF		0.10	I	BRVO
**Bolletta et al.**	50	M	Italy	Pfizer/BioNTech	2	DM		3	decreased VA	NA	0.04	intravitreal anti-VEGF		0.00	I	BRVO
**Bolletta et al.**	48	M	Italy	Pfizer/BioNTech	2	HTN		23	blurred vision	NA	0.00	intravitreal anti-VEGF		0.00	P	BRVO
**Bolletta et al.**	46	F	Italy	Pfizer/BioNTech	2			2	decreased VA	NA	0.30	oral prednisone		1.00	W	BRVO
**Cackett et al.**	41	F	United Kingdom.	AstraZeneca	1			5	blurring of vision	R	0.00	NA	NA	0.00	P	CRVO
**Cackett et al.**	49	F	United Kingdom.	AstraZeneca	N/A			4	blurring of vision	R	0.00	NA	NA	0.00	P	CRVO

**Casarini et al.**	60	M	Italy	Pfizer/BioNTech	2	ALL, DM	CMV-PORN, DR	1	blurring of vision	L	N/A		parsplana vitrectomy with silicone oil tamponade combined with cataract extraction was prescribed.	N/A	N/A	VH
**Che et al.**	87	F	South Korea	Pfizer/BioNTech	1	HTN		1	One day later, she developed sudden unilateral progressive visual loss in the right eye and scalp tenderness, and 4 days after the vaccination, she developed a fever of 38.3°C and visual loss in the left eye.	R	HM	intravenous high-dose methylpredprednisolone (250 mg per 6 hours) for 3 days followed by oral prednisolone (initial 60 mg/day for 2 weeks).		N/A	N/A	AAION
**Chen et al.**	48	F	Taiwan	AstraZeneca	1			28	inferior visual field	L	0.00	NA	NA	0.00	P	BRAO

									defects in the left eye							
**Choi et al.**	64	M	Korea	AstraZeneca	1			1	vision loss	R	0.10	observation with aspirin		N/A	N/A	CRVO
**Choi et al.**	33	F	Korea	Pfizer/BioNTech	2			6	vision loss	R	0.30	anti-VEGF injection		N/A	N/A	CRVO
**Choi et al.**	48	M	Korea	Pfizer/BioNTech	3			6	vision loss	R	0.80	anti-VEGF injection		N/A	N/A	CRVO
**Choi et al.**	69	F	Korea	AstraZeneca	1			3	vision loss	L	0.00	observation with aspirin		N/A	N/A	BRVO
**Choi et al.**	66	M	Korea	AstraZeneca	2			7	vision loss	L	0.00	observation		0.00	P	BRVO
**Choi et al.**	68	F	Korea	AstraZeneca	1		BRVO	1	vision loss	R	HM	observation		N/A	N/A	BRVO
**Choi et al.**	74	F	Korea	AstraZeneca	2	HTN, Nasal cavity cancer	BRVO	6	vision loss	R	HM		Vitrectomy	HM	P	BRVO
**Choi et al.**	63	F	Korea	AstraZeneca	1		CRVO	3	vision loss	L	1.50	anti-VEGF injection		1.50	P	CRVO
**Choi et al.**	62	M	Korea	AstraZeneca	1	HTN, DM, Cerebral infarction	ERM Secondary glaucoma	1	vision loss	L	0.50	observation		0.50	P	BRAO
**Chow et al.**	70	M	Taiwan	Moderna	1	HTN, Hyperlipidemia		5	acute painless vision loss in the right eye.	R	CF	clopidogrel and hyperbaric oxygen therapy to the patient	NA	N/A	N/A	CRAO
**Chung et al.**	65	F	Korea	AstraZeneca	2			15	sudden painless diminution of	R	CF	3-day course of intravenous methylprednisolone then 3-	NA	1.00	I	NAAION

									inferior visual field			week course of oral steroid.				
**Da Silva et al.**	27	F	Brazil	CoronaVac	2			14			N/A	N/A	N/A	N/A	N/A	CRAO
**Da Silva et al.**	57	F	Brazil	CoronaVac	2			15			N/A	N/A	N/A	N/A	N/A	CRAO
**Da Silva et al.**	84	F	Brazil	AstraZeneca	N/A	HTN, bilateral carotid atherosclerosis		16			N/A	N/A	N/A	N/A	N/A	CRAO
**Da Silva et al.**	74	F	Brazil	Moderna	N/A			3			N/A	N/A	N/A	N/A	N/A	CRAO
**Da Silva et al.**	39	F	Brazil	AstraZeneca	N/A	Psoriasis		3			N/A	N/A	N/A	N/A	N/A	CRAO
**Da Silva et al.**	66	F	Brazil	AstraZeneca	N/A	Hysterectomy		16			N/A	N/A	N/A	N/A	N/A	CRVO
**Da Silva et al.**	51	M	Brazil	Pfizer/BioNTech	N/A			6			N/A	N/A	N/A	N/A	N/A	CRVO
**Da Silva et al.**	66	M	Brazil	AstraZeneca	N/A	HTN		4			N/A	N/A	N/A	N/A	N/A	CRVO
**Da Silva et al.**	54	F	Brazil	AstraZeneca	N/A			10			N/A	N/A	N/A	N/A	N/A	CRVO
**Da Silva et al.**	56	F	Brazil	AstraZeneca	N/A			10			N/A	N/A	N/A	N/A	N/A	Intraretinal Hmg
**Da Silva et al.**	50	F	Brazil	AstraZeneca	N/A			15			N/A	N/A	N/A	N/A	N/A	Intraretinal Hmg
**Dutta Majumder & Prakash**	28	M	India	AstraZeneca	3			25	sudden onset painless	R	1.48	corticosteroid	N/A	0.18	I	CRVO

									loss of vision							
**Elhusseiny et al.**	51	M	USA	Pfizer/BioNTech	2	DM		1	sudden onset of blurred vision	L	CF	corticosteroid		1.30	I	NAAION
**Endo et al.**	52	M	Spain	Pfizer/BioNTech	1			15	sudden blurred vision	L	0.00	corticosteroid, bevacizumab, apixaban		0.00	P	CRVO
**Franco & Fonollosa**	53	M	Spain	Pfizer/BioNTech	2			7	visual loss	SD	L: 0.30, R: 0.00	acetazolamide		L: 0.30, R:0.00	P	NAAION
**Franco & Fonollosa**	65	M	Spain	Pfizer/BioNTech	1	HTN		12	blurry vision	R	1.00	observation		1.00	P	NAAION
**Girbardt et al.**	38	M	Multi	Pfizer/BioNTech	2	Ankylosing Spondylitis		3	painless visual field loss	R	1.00	acetylsalicylic acid, simvastatin		N/A	N/A	BRAO
**Girbardt et al.**	81	F	Multi	Pfizer/BioNTech	2	HTN		12	blurred vision	R	1.30	anti-VEGF		N/A	N/A	CRVAO
**Girbardt et al.**	40	M	Multi	Pfizer/BioNTech	1	DM, hypercholesterinemia		5	blurry vision, central grayish spot	L	0.10	N/A		1.25	W	venous stasis retinopathy
**Girbardt et al.**	67	M	Multi	AstraZeneca	1	ADHD		4	decreased vision, scotomata	R	1.00	N/A		N/A	N/A	NAAION
**Girbardt et al.**	32	M	Multi	Moderna	2			2	sudden scotoma	R	1.00	N/A		N/A	N/A	CRAO

**Girbardt et al.**	21	F	Multi	AstraZene ca	1			3	circumscri bed scotoma	SD	1.00	N/A		N/ A	N/A	AMN
**Goyal et al.**	28	M	India	Sputnik V	2			11	decreased VA	R	0.18	corticosteroid, apixaban		0.00	I	CRVO
**Ikegami et al.**	54	F	Japan	Moderna	2	Hypothyroidis m		2	sudden painless loss of vision	R	NLP	observation		N/ A	N/A	CRVAO
**Ishibashi et al.**	28	F	Japan	Pfizer/Bio NTech	1			15	visual field defects	R	-0.19	N/A		N/ A	N/A	BRAO
**Ishibashi et al.**	86	M	Japan	Pfizer/Bio NTech	2	HTN		4	visual field defects	L	0.10	N/A		N/ A	N/A	BRAO
**Ishibashi et al.**	62	M	Japan	Pfizer/Bio NTech	2			7	visual field defects	L	-0.07	N/A		N/ A	N/A	PAMM
**Ishibashi et al.**	33	F	Japan	Pfizer/Bio NTech	2	HTN, EDRD, Alport Syndrome		8	visual field defects	L	0.16	N/A		N/ A	N/A	AMN
**Ishibashi et al.**	80	M	Japan	Pfizer/Bio NTech	2	HTN, DM		42	N/A	R	0.00	N/A		N/ A	N/A	BRAO
**Ishibashi et al.**	57	F	Japan	Pfizer/Bio NTech	2			61	N/A	R	-0.19	N/A		N/ A	N/A	BRAO
**Kang et al.**	64	M	Korea	Pfizer/Bio NTech	1	HTN, DM	cataract surgery	3	sudden painless loss of vision, visual field defects	R	0.40	aspirin, atorvastatin calcium trihydrate, brimonidine tartrate, dorzolamide/ti molol	anterior chamber paracentes is	0.00	I	BRAO
**Lee et al.**	34	M	USA	Pfizer/Bio NTech	2			11	Blurring of vision	L	CF	intravitreal aflibercept,		0.18	I	CRVAO

												corticosteroids, bromfenac, and acetazolamide, pentoxifylline				
**Li et al.**	55	F	China	CoronaVac	1	HTN, DM		1	N/A	L	1.00	N/A		N/A	N/A	BRAO
**Li et al.**	33	M	China	CoronaVac	3	HTN,DM		7	N/A	R	1.00	N/A		N/A	N/A	BRVO
**Li et al.**	71	M	China	CoronaVac	1		DR, Pan-retinal photocoagulation	<1	N/A	R	1.30	N/A		N/A	N/A	CRAO
**Li et al.**	54	M	China	CoronaVac	1	HTN, DM		8	N/A	R	LP	N/A		N/A	N/A	CRVO
**Li et al.**	58	M	China	CoronaVac	1		DR, Pan-retinal photocoagulation	<1	N/A	R	CF	N/A		N/A	N/A	VH
**Lin et al.**	61	F	Taiwan	AstraZeneca	1	HTN, Hyperlipidemia	cataract surgery	7	Blurring of vision, temporal headache	L	0.40	corticosteroid		0.60	W	NAAION
**Maleki et al.**	79	F	USA	Pfizer/BioNTech	2	Osteoperosis		35	sudden bilateral loss of vision	SD	R: 1.80, L:0.30	SC tocilizumab		N/A	N/A	AAION
**Maleki et al.**	33	F	USA	Moderna	2			10	progressive nasal field defect, photopsia	L	0.00	azathioprine, cyclosporine		N/A	N/A	AZOOR

**Murgova & Balchev**	45	M	Bulgaria	Pfizer/BioNTech	2			25		L	1.00	Vasodilators, Anti-platelet		1.00	P	NAAION
**Nachbor et al.**	64	F	Nepal	Pfizer/BioNTech	1	DM	cystoid macular edema CME, Cataract surgery	6	sudden painless loss of vision	L	0.60	N/A		N/A	N/A	NAAION
**Nusanti et al.**	50	F	Indonesia	CoronaVac	1	graves diseases		2	acute proptosis, decreased vision, ophthalmoplegia	SD	R: LP, L: 1.17	IV corticosteroids, SC daparinux		0.00	I	N/A
**Park et al.**	82	F	Korea	Pfizer/BioNTech	1	HTN, DM, Hypothyroidism, Syphillis	AMD, 53 Anti-VEGF injections (Last 7 days before)	3		SD	R: 0.2, L:0.04	Observation		R: 0.2, L:0.04	P	Ssubmacular hemorrhage
**Park et al.**	81	M	Korea	Pfizer/BioNTech	1	HTN, Atrial fibrillationCAOD, Spinal stenosis, HCMP	Cataract surgery	10		R	0.40		C3F8	CF	W	submacular hemorrhage
**Park et al.**	81	M	Korea	Pfizer/BioNTech	2		cataract surgery	6		L	0.02		C3F8	CF	W	submacular hemorrhage
**Park et al.**	82	F	Korea	Pfizer/BioNTech	1	DM, HTN, Angina	Cataract surgery, Vitrectomy for vitreous	3		R	0.15	anti-VEGF (Bevacizumab), C3F8	C3F8	0.04	I	submacular hemorrhage

							hemorrhage									
**Park et al.**	84	F	Korea	Pfizer/BioNTech	2	Polycystic kidney	AMD23 Anti-VEGF injections (Last 5 months before)	15		L	0.10	anti-VEGF (Beva cizumab), C3F8	C3F8	0.10	P	submacular hemorrhage
**Park et al.**	77	F	Korea	Pfizer/BioNTech	1	HTN, HBV carrier, Sigmoid colon cancer on chemotherapy, s/p low anterior resection	Cataract surgery	16		R	N/A	R: Anti-VEGF (Ranibizumab), L: Anti-VEGF (Beva cizumab)		R: 0.2, L: 0.8	N/A	submacular hemorrhage
**Park et al.**	70	M	Korea	NA	1	DM	AMD, 13 Anti-VEGF injections (Last 4, months before)	28		R	0.15	anti-VEGF (Bevacizumab)		0.04	I	submacular hemorrhage
**Park et al.**	82	M	Korea	Pfizer/BioNTech	1	HTN, DM	PCV, Cataract surgery26 Anti-VEGF injections (Last 7 months before), Photodynamic therapy	14		SD	0.05	anti-VEGF (Bevacizumab)		0.04	I	RVO

**Park et al.**	71	F	Korea	AstraZeneca	1	Dyslipidemia	AMD, 2 Anti-VEGF injections (Last 6 months before)	2		R	0.60	anti-VEGF (Bevacizumab)		0.30	I	submacular hemorrhage
**Park et al.**	80	F	Korea	Pfizer/BioNTech	1	HTN, DM	AMD, 12 Anti-VEGF injections (Last 15 months before)	28		R	0.04	anti-VEGF (Bevacizumab)		0.04	P	submacular hemorrhage
**Park et al.**	62	M	Korea	AstraZeneca	1	DM		28		R	0.30	observation		0.30	P	submacular hemorrhage
**Park et al.**	68	F	Korea	AstraZeneca	1	Dyslipidemia	Vitrectomy for vitreous hemorrhage, Cataract Surgery	1		L	N/A	observation		HM	N/A	RVO
**Park et al.**	76	M	Korea	Pfizer/BioNTech	1	HTN	NTG, Cataract surgery	3		L	1.00	anti-VEGF (Bevacizumab)		0.80	I	RVO
**Park et al.**	85	F	Korea	Pfizer/BioNTech	2	DM, HTN, ESRD, Old tuberculosis, Dementia	Vitrectomy with cataract surgery for vitreous	1		R	0.10	Observation		CF	W	RVO

							hemorrhage									
**Park et al.**	59	M	Korea	AstraZeneca	1	HTN, DM	Vitrectomy with secondary IOL scleral fixation	2		L	0.80	anti-VEGF (Bevacizumab)		0.80	P	RVO
**Park et al.**	61	M	Korea	AstraZeneca	1			2		R	N/A	anti-VEGF (Bevacizumab)		0.04	N/A	RVO
**Park et al.**	79	M	Korea	Pfizer/BioNTech	2	DM, Early gastric cancer		2		L	N/A	anti-VEGF (Bevacizumab)		0.40	N/A	RVO
**Park et al.**	63	M	Korea	Pfizer/BioNTech	1	DM	DME, Intravitreous triamcinolone injection	13		R	0.40	anti-VEGF (Bevacizumab)		0.01	I	RVO
**Park et al.**	51	F	Korea	AstraZeneca	1	HTN		21		L	N/A	anti-VEGF (Bevacizumab)		0.09	N/A	RVO
**Park et al.**	81	F	Korea	Pfizer/BioNTech	1	HTN	Cataract surgery	4		L	N/A	observation		0.30	N/A	RVO
**Park et al.**	61	M	Korea	AstraZeneca	1	HTN	Uveitis	3		L	1.00	observation		0.90	I	RVO
**Peters et al.**	71	M	Australia	AstraZeneca	1			2	reduced visual acuity	NA	1.00	anti-VEGF (Bevacizumab)	NA	N/A	N/A	BRVO
**Peters et al.**	58	M	Australia	AstraZeneca	1		pterygium excision	3	reduced visual acuity	NA	0.48	anti-VEGF (Bevacizumab)	NA	N/A	N/A	RVO

**Peters et al.**	73	F	Australia	AstraZeneca	1	HTN	macula-off RRD, macula hole, PCIOL OU	3	reduced visual acuity	NA	0.50	antiVEGF (Aflibercept)	NA	N/A	N/A	BRVO
**Peters et al.**	47	F	Australia	Pfizer/BioNTech	1	Hyperthyroidism		5	reduced visual acuity	NA	0.20	anti-VEGF (Bevacizumab)	NA	N/A	N/A	BRVO
**Peters et al.**	36	M	Australia	Pfizer/BioNTech	2			2	reduced visual acuity	NA	0.18	anti-VEGF (Aflibercept)	NA	N/A	N/A	CRVO
**Priluck et al.**	57	F	USA	Moderna	2			21	flashes and floaters	L	0.00	anti-VEGF (Aflibercept)	NA	0.00	P	BRVO
**Priluck et al.**	20	F	USA	Johnson & Johnson	N/A			8	nd myopia presented with 1 week of new,	SD	0.00	abstain from coffee and oral birth control for the time-being	NA	0.00	P	AMN
**Pur et al.**	34	M	Canada	Johnson & Johnson	1			2	He reported blurriness in the inferior visual field in his right eye as well as intermittent photopsia	R	0.00		NA	0.00	P	BRVO
**Romano et al.**	54	F	Italy	AstraZeneca	2	HTN		2	15-day history of unilateral,	R	1.00		NA	1.00	P	CRVO

									painless loss of vision in her right eye (RE).							
**Sacconi et al.**	74	F	Italy	Moderna	2	AF		2	painless vision loss in the right eye experienced 48 hours	R	0.30	observation for 3 weeks then two injections of intravitreal ranibizumab		0.30	P	RVO
**Sanjay et al.**	50	F	India	AstraZeneca	1			4			N/A			N/A	N/A	N/A
**Shah et al.**	27	F	USA	Pfizer/BioNTech	2	polycystic ovarian syndrome, Idiopathic Intracranial HTN, Anemia		10	floater and wavy lines OS	L	0.00	3 doses of intervitreal injection (ranibizumab)		0.00	P	CRVO
**Sodhi et al.**	43	M	India	AstraZeneca	1			3	sudden painless drop of vision OS	L	R: 0.2, L: 1.5	intravitreal injection of triamcinolone acetonide (4 mg/0.1 ml) + targeted laser		R: 0.2, L: 1.5	P	CRVO
**Sonawane et al.**	50	M	India	AstraZeneca	2	DM, renal impairment		4	right eye diminution of vision	R	0.00	Anti-VEGF injection		N/A	N/A	CRVO
**Sonawane et al.**	43	F	India	AstraZeneca	2			3	Right eye sudden-onset diminution of vision	R	1.08	Observation		N/A	N/A	CRVO

**Sugihara et al.**	38	M	Japan	Pfizer/BioNTech	2			15	decreased vision OS	L	N/A	2 doses of anti-VEGF (Aflibercept)	NA	1.20	N/A	BRVO
**Takacs et al.**	35	M	Hungary	mRNA (unspecified)	1			12	decreased vision	R	N/A	ASA, reduced smoking and single dose of intravitreal anti-VEGF (aflibercept)	ASA	N/A	N/A	CRVO
**Tanaka et al.**	71	F	Japan	Pfizer/BioNTech	2		inferior temporal BRVO and secondary macular edema	1	vision loss	L	0.18	anti-VEGF (Aflibercept)		0.00	I	BRVO
**Tanaka et al.**	74	M	Japan	Pfizer/BioNTech	1		temporal superior BRVO	1	vision loss	R	0.10	2 doses of intravitreal ranibizumab		0.00	I	BRVO
**Thammakumpee et al.**	41	M	Thailand	AstraZeneca	2			7	blurred vision		N/A	NA		0.70	N/A	CRAO
**Tsukii et al.**	55	F	Japan	Pfizer/BioNTech	1			7	OD inferior visual field disturbance		0.00	NA		0.00	P	NAAION
**Vinzamuri et al.**	35	M	India	AstraZeneca	1			30	blurring of vision, black spots in vision, and reduced brightness of vision	SD	N/A	NA		N/A	N/A	N/A

**Vujosevic et al.**	69	F	Italy	AstraZeneca	1	DVT		7	vision loss	R	0.20		Laser photocoagulation	0.00	I	BRVO
**Vujosevic et al.**	82	F	Italy	Pfizer/BioNTech	2			14	vision loss	R	0.50	Steroid treatment		0.30	I	BRVO
**Vujosevic et al.**	96	F	Italy	Pfizer/BioNTech	2	HTN, DM		7	vision loss	R	1.00	Steroid treatment		1.00	P	CRVO
**Vujosevic et al.**	91	F	Italy	Pfizer/BioNTech	2			10	vision loss	L	CF	Observation due patient refused treatment		CF	P	CRVO
**Vujosevic et al.**	78	F	Italy	Pfizer/BioNTech	2			7	vision loss	R	0.10	Anti-VEGF agents		0.00	I	BRVO
**Vujosevic et al.**	70	M	Italy	AstraZeneca	1			7	vision loss	R	0.00	None		0.00	P	CRVO
**Vujosevic et al.**	40	M	Italy	AstraZeneca	1	Hyperhomocysteinemia		14	vision loss	R	0.00	None		0.00	P	BRVO
**Vujosevic et al.**	76	M	Italy	AstraZeneca	1	HTN		42	vision loss	R	CF	None		CF	P	CRAO
**Vujosevic et al.**	91	M	Italy	Pfizer/BioNTech	2	DM		28	vision loss	R	0.20	Steroid treatment		0.20	P	BRVO
**Vujosevic et al.**	72	F	Italy	Pfizer/BioNTech	2	HTN, HL		21	vision loss	R	0.10	Steroid treatment		0.00	I	BRVO
**Vujosevic et al.**	88	M	Italy	Pfizer/BioNTech	2	HTN, HL, CVD, Alzheimer, K Prostate		14	vision loss	R	0.80	Steroid treatment		0.80	P	RVO
**Vujosevic et al.**	73	F	Italy	AstraZeneca	2	HTN, HL, CVD, NET		28	vision loss	R	CF	Steroid treatment		CF	P	CRVO

**Vujosevic et al.**	65	F	Italy	Johnson & Johnson	1	HTN, DM, HL		7	vision loss	R	0.30	Steroid treatment		0.20	I	CRVO
**Vujosevic et al.**	72	F	Italy	AstraZeneca	1	HTN, CVD		14	vision loss	L	0.40		Laser photocoagulation	0.40	P	HRVO
**Wang et al.**	70	M	Taiwan	Moderna	2			5	vision loss	R	CF	hyperbaric oxygen therapy (HBOT)		CF	P	CRAO
**Elnahry et al.**	69	F	Egypt	Pfizer/BioNTech	2			16	visual loss	SD	N/A	Steroid treatment		N/A	I	AAION
**Elnahry et al.**	32	F	Egypt	AstraZeneca	1			6	visual loss	L	N/A	Steroid treatment		N/A	I	AAION
**Haseeb et al.**	40	M	USA	Pfizer/BioNTech	1	DM		5	visual loss	L	0.3	Aspirin		N/A	I	AAION
